# Mechanical characterization of rose bengal and green light crosslinked collagen scaffolds for regenerative medicine

**DOI:** 10.1093/rb/rbab059

**Published:** 2021-11-02

**Authors:** Joy Braun, Stefanie Eckes, Michelle Fiona Kilb, Dirk Fischer, Claudia Eßbach, Pol Maria Rommens, Philipp Drees, Katja Schmitz, Daniela Nickel, Ulrike Ritz

**Affiliations:** 1 Department of Orthopedics and Traumatology, BiomaTiCS, University Medical Center, Johannes Gutenberg University, Langenbeckstraße 1, Mainz 55131, Germany; 2 Clemens-Schöpf-Institute of Organic Chemistry and Biochemistry, Technical University of Darmstadt, Alarich-Weiss-Straße 4, Darmstadt 64287, Germany; 3 Berufsakademie Sachsen—Staatliche Studienakademie Glauchau, University of Cooperative Education, Kopernikusstraße 51, Glauchau 08371, Germany

**Keywords:** collagen type I, collagen laminates, rose bengal and green light crosslinking, thickness analysis, micro tensile testing, cell–collagen interactions

## Abstract

Collagen is one of the most important biomaterials for tissue engineering approaches. Despite its excellent biocompatibility, it shows the non-negligible disadvantage of poor mechanical stability. Photochemical crosslinking with rose bengal and green light (RGX) is an appropriate method to improve this property. The development of collagen laminates is helpful for further adjustment of the mechanical properties as well as the controlled release of incorporated substances. In this study, we investigate the impact of crosslinking and layering of two different collagen scaffolds on the swelling behavior and mechanical behavior in micro tensile tests to obtain information on its wearing comfort (stiffness, strength and ductility). The mechanical stability of the collagen material after degradation due to cell contact is examined using thickness measurements. There is no linear increase or decrease due to layering homologous laminates. Unexpectedly, a decrease in elongation at break, Young’s modulus and ultimate tensile strength are measured when the untreated monolayer is compared to the crosslinked one. Furthermore, we can detect a connection between stability and cell proliferation. The results show that with variation in number and type of layers, collagen scaffolds with tailored mechanical properties can be produced. Such a multi-layered structure enables the release of biomolecules into inner or outer layers for biomedical applications.

## Introduction

Tissue Engineering (TE) is a promising approach for the repair of musculoskeletal defects like tendon rupture, muscle atrophy, lesions or bone fractures that still pose challenging clinical problems [1]. Therefore (natural) bioactive biomaterials acting as scaffolds and drug carriers are needed. Collagen is the most abundant protein in the body and often used in TE due to its excellent properties. It is highly biocompatible, being a significant component of the extracellular matrix, has well-tolerated degradation products and additionally negligible immunogenicity [2–4]. Nevertheless, there are some limitations in its clinical use due to its high swelling degree, rapid biodegradation and its low biomechanical stiffness [5–9]. Modification of collagen by reaction of its functional groups allows the creation of materials with tailored properties. There are different concepts for covalent crosslinking of collagen. The most commonly used chemical crosslinking method uses glutaraldehyde (GA). It is well known to improve the mechanical strength, but it is also associated with cytotoxicity after treatment as a result of toxic residues [10–12]. Another possibility to modify collagen is the use of 1-ethyl-3(3-dimethylamino) propyl carbodiimide and N-hydroxysuccinimide (EDC/NHS) [13]. The products of crosslinking are biocompatible and more stable; however, the process is very time-consuming [14–16].

A fast and simple alternative is photochemical crosslinking by rose bengal and green light (RGX), which is approved for several clinical applications [17–19]. Chan and So showed that the treatment of collagen with RGX improves thermostability and mechanical properties [6]. Additionally, it was possible to prolong the initial burst release of proteins in photochemically crosslinked collagen structures [7, 20]. Further information about the influence of photochemical crosslinking on the stability and mechanical properties of collagen sheets is necessary. By including cells biomaterial–cell interactions can be defined since cell behaviors such as adhesion and growth are affected for example by the Young’s modulus [21, 22].

In a former study, we investigated the influence of RGX treatment on two different collagen scaffolds as well as the effect of multilayered collagen structures called laminates and could show that we were able to produce RGX crosslinked collagen laminates, which are—due to their modulate structure—attractive for medical applications. In our preliminary experiments regarding thickness, swelling ratio, drug release and cell behavior in commercially available collagens, we were able to load the scaffolds with desired concentrations of biomolecules [9].

In this study, we investigated the mechanical characteristics (Young’s modulus, tensile strength and elongation at fracture point) of two commercially available collagens and different composed multilayered collagen laminates by micro tensile testing. The analysis of the mechanical stability and the microstructure of these collagens was completed by the determination of the swelling degree and the surface topography by the confocal laser scanning microscopy (CLSM). Herein, a comparison of untreated and RGX crosslinked collagens was performed as well as the analysis of the differently composed laminates. As fibroblasts are the main cell type in connective and other tissues [23], we further analyzed their influence on the thickness of the collagen scaffolds and therefore their mechanical stability and destabilization after contact with cells as well as their proliferation capacity on the laminates. The overriding goal is to develop multilayer laminates (>3 layers) with incorporated biomolecules in each layer, for a controlled release in medical applications. Therefore the aim was to gain a deeper insight into the relationships between structure, treatment and mechanical properties of the selected collagens and their laminates since these are the parameters that influence cell behavior and release kinetics. To conclude the study, the influence of RGX treatment on two different biomolecules was tested since photochemical crosslinking is part of the laminate production. Therefore stromal-derived factor 1α (SDF-1α) and bone morphogenetic protein 7 (BMP-7) were chosen as model factors for an example in medical applications of the laminates in bone regeneration. Since bone is highly vascularized, SDF-1α plays an important role in bone regeneration. It is known to play a crucial role in angiogenesis through the activation, migration and proliferation of endothelial cells and could therefore improve bone healing [24, 25]. To test whether SDF-1α is still active after crosslinking the proliferation of endothelial cells was investigated. BMPs, belonging to the transforming growth factor-β superfamily, are known for their osteogenic properties through promoting the proliferation and differentiation of osteogenic cells [26, 27]. Here we test the osteoblastic differentiation potential under influence of BMP-7. With this huge bandwidth of findings, more insights can be achieved regarding healing capacity and the controlled release of molecules in the different layers.

## Materials and methods

### Collagen sheets

Two commercially available collagen samples were used: a non-perforated collagen film (Collagen Solutions/C) from Collagen Solutions (London, UK) and an Atelocollagen sponge (A) (CLS-01, Koken Co. Ltd, Tokyo, Japan), which is a highly pure porous, sponge-like collagen derived from bovine dermis that will be referred to in the following as Atelocollagen/A.

### Collagen photo crosslinking and laminate preparation

For thickness analyses, proliferation assays, cytotoxicity testing and activity testing, collagen samples were cut into circles with a diameter of 1 cm. Rectangles (0.5 × 2 cm^2^) were used for micro tensile testing which were cut into 0.5 × 1 cm^2^ samples for the analysis. For crosslinking, the collagen sheets were loaded with 0.01% rose bengal (RB from Alfa Aesar, Haverhill, Massachusetts, USA) in phosphate-buffered saline (PBS, Gibco^®^ Invitrogen™ Life Technologies, Carlsbad, USA). The used volume corresponded to 100% of the swelling capacity determined for a 1 × 1 cm^2^ collagen piece in swelling experiments as described earlier [9]. For crosslinking, single sheets were exposed for 10 min (from one side) to green light (λ_max_ = 565 nm) using a mounted LED (M565L3, Thorlabs, Bergkirchen, Germany). The distance between the light source and the samples was 2 cm, so that an irradiance of 45 mW was achieved. To prepare collagen laminates, an additional coating of 20 µl RB (referring to a size of 1 × 1 cm) was placed between all collagen layers whereas the light exposure time remained the same. For all cell experiments, collagen samples were sterilized before crosslinking. For this purpose, the round collagen sheets with a diameter of 1 cm were equilibrated in PBS for 2 h, sterilized for 45 min under UV light and placed in a 48-well plate for drying overnight. Crosslinked collagen monolayers were further termed with RGX after the collagen abbreviation. Bi- and multilayer laminates are named by listing the collagen abbreviations. CAC is for example a multilayer laminate composed of a layer of C followed by an A and a C layer.

### Confocal laser scanning microscope

Dry collagen sheets were examined via confocal laser scanning microscope (LEXT OLS5000 by Olympus Deutschland GmbH, Hamburg, Germany) with a magnification of 50×. Images were recorded in the stitching modus with an area size of 4 × 4 for A and 5 × 5 for C Solutions.

### Micro tensile testing

Micro tensile tests were conducted at room temperature with a tensile (compression) stage (Kammrath & Weiss GmbH, Dortmund, Germany). The sample size was 0.5 × 0.1 cm^2^ with a gauge length of 1 mm. After equilibrating the samples for 10–15 min in PBS, the soaked samples were placed in the cavity of the gripping device of the micro tensile module and fixed mechanically by means of a screw fitting with terminal blocks. For Collagen Solution, additional sandpaper was used at the top and the bottom of the sample holder allowing a convenient fixation. The micro tensile tests were performed under displacement control (10 µm/s). The load was measured using a load cell of maximum 1000 mN and elongation by means of a linear variable differential transformer. The maximum travel of the tensile stage is limited to 5 mm unilaterally. Measurements continued until the maximum of the load cell or the maximum of travel was reached or a rupture of the test specimen occurs. The system control and data acquisition were based on a deformation device system. Alignments were realized by gauge block to guarantee a uniaxial state of stress. For C a pre-displacement, i.e. an initial displacement, is necessary to realize alignment of the microstructure between the bearing ends of the specimens and the grips. To calculate stress–strain curves from the measured load-elongation data, length and width as well as thickness of the specimens are needed. All conventional methods for thickness measurements were omitted due to the sample condition. In this study, the determination of the thickness was conducted using the height gauge (DIGIMAR CX1-DX1, Mahr, Göttingen, Germany). After equilibrating the samples in PBS for 10 min, the sample was compressed in PBS with a defined force of 1 ± 0.2 N. The value obtained corresponds approximately to the thickness. Elongation at break, Young’s modulus and ultimate tensile strength were calculated from the obtained stress–strain curves. Micro tensile testing was performed for each sample up to 3 times with the following samples: C, A, C_RGX_, A_RGX_, CC, AA, CCC and AAA.

### Swelling degree

The analysis of the swelling degree was performed as described previously [9]. Briefly, the dry weight (*m*_d_) of the collagen sample (1 × 1 cm) was determined by weighing each sample 3 times. The collagen samples were incubated in 2 ml of PBS at room temperature for 10 min. The wet collagen samples were weighed 3 times each after blotting the non-absorbed liquid to determine the wet weight (*m*_w_). The swelling degree was calculated according to the following equation:

$$\mathrm{swelling}\;\mathrm{degree}=\;\frac{m_{w}-m_{d}}{m_{d}}\;x\;100\%$$

The experiment was performed 3 times and each sample was weighed 3 times. Swelling degree was determined for the following samples: C, C_RGX_, CC, CCC, A, A_RGX_, AA and AAA.

### Cell culture

Normal human dermal fibroblasts (NHDF, PromoCell, Heidelberg, Germany) and L929 mouse fibroblasts (ATCC, Manassas, Virginia, USA) were maintained in Dulbeccós Modified Eagle Medium + GlutaMAX (Gibco, Life Technologies, Grand Island, NY, USA) containing 10% fetal calf serum (FCS from Biochrom AG, Berlin, Germany), 100 U/ml penicillin and 100 µg/ml streptomycin sulfate (Sigma-Aldrich^®^GmbH, St. Louis, MO, USA). The cells were incubated in a humidified atmosphere (5% CO_2_, 37°C) and split at 80% of confluence.

Primary cultures of human umbilical vein endothelial cells (HUVEC) were purchased from PromoCell (Heidelberg, Germany). Cells were cultured in endothelial cell growth medium MV (PromoCell, Heidelberg, Germany) and supplemented with the reagents provided in the kit.

Bone fragments from hip replacement were gained from three different patients and used for the isolation of primary human osteoblasts (hOB). Informed consent was obtained from all patients and the local ethics committee approved the investigations. Isolation followed a previously described protocol [28, 29]. In brief, bone specimens were eliminated from attached fibrous and fat tissue. After a vigorous rinse in PBS, the fragments were digested with collagenase type IV (Sigma Aldrich ^®^ GmbH, St. Louis, USA) for 45 min at 37°C with a subsequent PBS washing step. Bone specimens were cut into 2 mm pieces and three of them were placed in a 6-well plate. For cultivation DMEM/F12 (Biochrom, Berlin, Germany) supplemented with 10% FCS (PAA Lab, Austria), 100 U/ml penicillin, 100 µg/ml streptomycin sulfate (Sigma-Aldrich GmbH, St. Louis, MO, USA) were used.

### Thickness analysis

For thickness analysis, a total number of 10 000 cells/well were seeded onto the sheets in 24 well culture dishes and incubated for 24 and 72 h. Afterwards, the collagen sheets and laminates were removed, washed in PBS, fixed for 15 min in 3.7% paraformaldehyde in PBS and dried at room temperature. Dry samples were equilibrated for 10 min in PBS before thickness was analyzed via height gauge (DIGIMAR CX1-DX1, Mahr, Göttingen, Germany) at room temperature. The samples were subjected to a force of 1 ± 0.2 N exerted on a circular area with a diameter of 1 cm. The debouncing time was set to 5 s and the probing speed was 3 mm/s. Thickness analysis was performed for each sample at least twice with a minimum of three repeated measurements. The thickness values serve as a measure for mechanical stability after destabilization due to cell contact. The following samples were analyzed: C, A, C_RGX_, A_RGX_, CC, AA, AC, CCC, AAA and CAC.

### Cell viability on collagen laminates

Proliferation of NHDF cells on the laminates was tested with alamarBlue^®^. To get a result that is comparable with the thickness analysis, the same amount of cells was used (10 000 cells/well) and the alamarBlue^®^ assay was performed 24 and 72 h after seeding. Cells were incubated for 4 h at 37°C with 320 µl of a 10% alamarBlue^®^ solution. Subsequently, 3 × 100 µl of the supernatant were transferred into a 96-well plate. The fluorescence intensity was measured with the GloMax^®^Multidetection System (excitation 525 nm; emission 580–649 nm). Cell viability tests on laminates were performed 3 times independently in duplicates with the following samples: C, A, C_RGX_, A_RGX_, CC, AA, AC, CCC, AAA and CAC.

### *In-vitro* cytotoxicity testing of the laminates

For *in-vitro* cytotoxicity testing of the laminates L929 cells were used. Ten thousand cells/well were seeded in a 96-well plate for 24 h. Simultaneously, laminates were incubated with cell culture medium. After 24 h the L929 cell medium was replaced by the medium that had been incubated with laminates and the cells were incubated for 24 h. Subsequently, 100 µl/well of a 1 mg/ml 3-(4,5-dimethylthiazol-2-yl)-2,5-diphenyltetrazolium Bromide (MTT) (Sigma-Aldirch^®^GmbH, St. Louis, MO, USA) solution in serum-free medium was added and incubated for 2 h before the solution was decanted. A measured quantity of 100 µl/well isopropanol (Thermo Fisher Scientific, Waltham, MA, USA) was added and the absorption was measured (570/560 nm). *In-vitro* cytotoxicity of the laminates was determined 3 times independently in duplicates with the following samples: C, A, C_RGX_, A_RGX_, CC, AA, AC, CCC, AAA and CAC.

### Activity of different biomolecules after crosslinking

To test whether crosslinking affects the bioactivity of incorporated biomolecules, functional assays were performed after the molecules were crosslinked with the Collagen membranes.

#### Molecule adsorption

BMP-7 (Miltenyi Biotec B.V. Co. KG, Bergisch Gladbach, Deutschland) and SDF-1α (Peprotech Inc. Rocky Hill, SC, USA) were diluted in RB to get the desired concentration (BMP-7 100/500 ng, SDF-1α 500 ng). Since this leads to a smaller RB concentration, a higher RB concentration was used to get a final concentration of 0.01% on the sheets. After 2 h of swelling, crosslinking occurred.

##### Activity of SDF-1α after crosslinking

The alamarBlue^®^ assay was used to test the proliferation of HUVECs under the influence of SDF-1α, which was an indirect method to test whether SDF-1α is still active after crosslinking. Therefore 7.500 HUVECs/well were used and the test was performed as described previously on Day 3.

##### Activity of BMP-7 after crosslinking

Alkaline phosphatase (ALP) assay was used to determine the osteoblast mineralization capacity under influence of BMP-7 after crosslinking on the collagen sheets. ALP is a specific marker for the assessment of osteogenic differentiation [30]. BMP-7 activity testing was performed with primary osteoblasts. Therefore collagens were placed as mentioned above in a 48-well plate and 100 000 cells/well were used. Samples were incubated for 6 days with a media change on Day 3 before the ALP assay was performed. Wells were washed twice with PBS and incubated for 30 min at 37°C with 250 µl of 0.1%Triton X-100 (Sigma-Aldrich^®^GmbH, St. Louis, MO, USA) in aqua bidest. Thereupon, 200 µl of p-nitrophenylphosphate (Sigmal-Aldirch^®^GmbH, St. Louis, MO, USA) was added with an additional incubation of 30 min. NaOH (50 µl) was used to stop the reaction and 3 × 100 µl per sample were transferred to a 96-well plate. The absorbance was measured at 405/700 nm and a calibration curve was used to determine the final concentration. Experiments were repeated 3 times.

### Statistical analysis

Statistical analysis was performed with the SPSS software Version 24 (IBM, Endicott, NY, USA) and values are presented as mean values ± standard deviation. For statistical analysis, a one-way analysis of variance was used. Tukey *post hoc* analysis was performed when homogeneity of variance was ensured (Levene’s test, *P* > 0.05). When homogeneity was excluded (Levenés test, *P* < 0.05) Games–Howell *post hoc* analysis was performed. A *P* value of < 0.05 is assumed as statistically significant.

## Results

### Impact of crosslinking on surface topography

**Figure 1.**
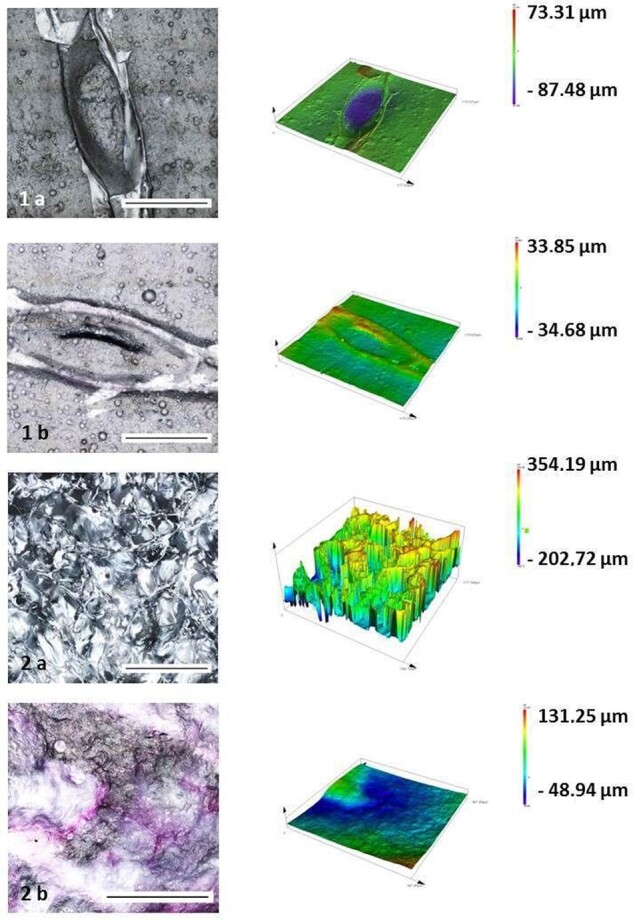
Confocal laser scanning microscope images of Collagen solution (1) and Atelocollagen (2) before **(a)** and after **(b)** RGX treatment. Magnification of 50×, scale bar 100 µm.

Surface topography before and after RGX treatment was investigated with CLSM. Regarding the macroscopic perspective of the topography, Collagen Solutions (C) seemed to be relatively dense with visible imprints, whereas A showed an open, sponge-like structure (Fig. 1). Crosslinking by RGX treatment resulted in changes in the surface topography of both materials. For C, crosslinking leads to a leveling of the imprints near the level of the remaining collagen. The surface of the remaining collagen itself was only slightly affected. In contrast, A showed a total collapse of the sponge-like structure. These findings are in line with earlier results from our group which showed a total collapse of A after PBS contact [9].

### Impact of crosslinking and layering on mechanical behavior

Micro tensile tests were performed to characterize the mechanical behavior: in particular ductility and stiffness, of the collagen sheets to study the impact of RGX and layering on the mechanical behavior, laminates were also investigated. The samples listed in Table 1 were tested. Due to the complexity of measurement only homogenous/single-material laminates were investigated.

**Table 1.** Used collagen samples for micro tensile testing

Monolayer	Bilayer	Multilayer
A _(untreated)_	AA	AAA
A _(RGX)_	CC	CCC
C _(untreated)_		
C _(RGX)_		

**Figure 2.**
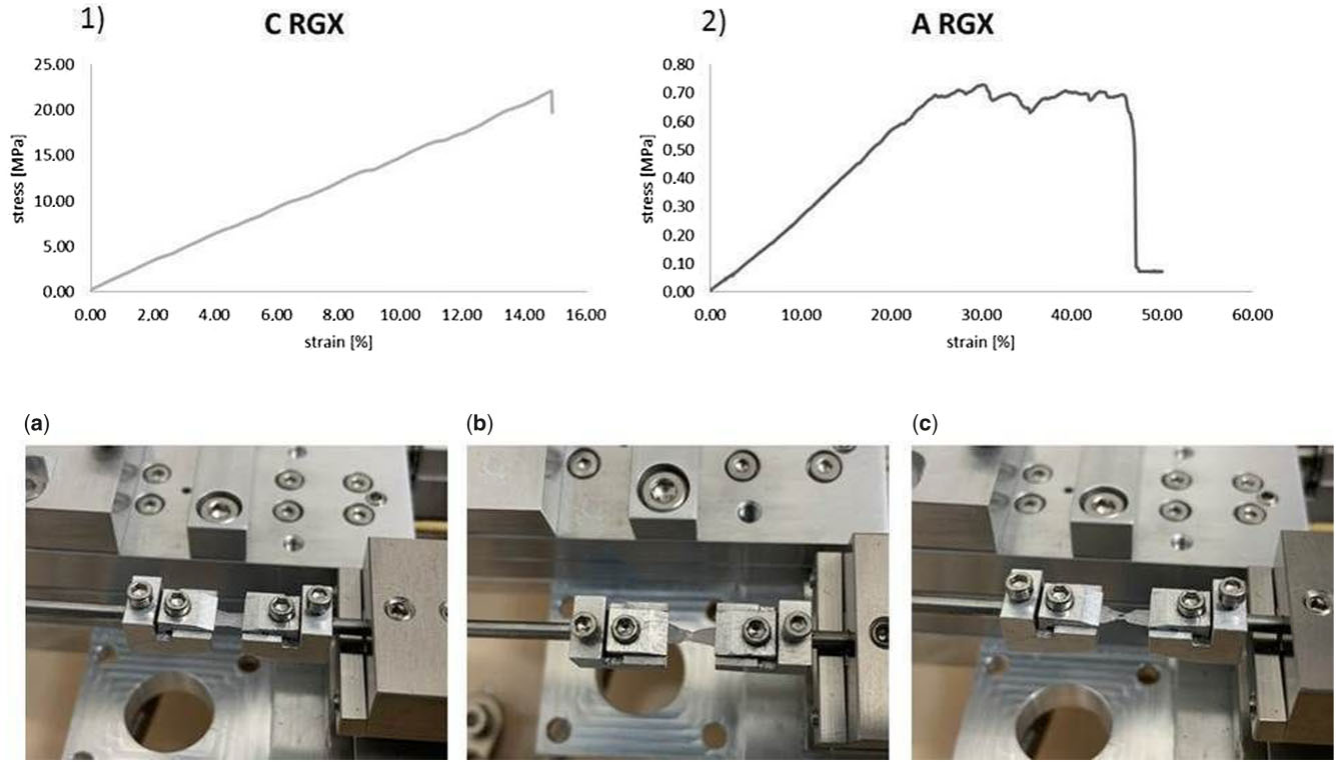
Typical stress**–**strain curves for C (1) and A (2) and sequence **(a)** to **(c)** show elongation and ductile failure during micro tensile testing.

Figure 2 (1 and 2) shows the typical stress–strain curves for C and A. The fundamental course of the curve is independent of treatment. For the calculation of the Young’s modulus of C two different curve progressions were observed (Supplementary Material). Certain data were averaged for the calculation since the real behavior might be somewhere in between.

Both, A and C, collagen materials show a mismatch of mechanical behavior particularly in the near-zero stress and strain, where the stress–strain curve is linear. In contrast to A, where the end of the curve is reached through the failure of the material, the curve of C ends with the linear part when the maximum strain is reached. That means, the material undergoes reversible elastic deformation and the material returns to its original shape after the load is removed. The relationship between stress and strain is described by Hooke's law. The slope of the stress–strain curve is called Young’s modulus and is a mechanical property that measures the tensile stiffness of a solid material. The higher the Young’s modulus, the more stress is needed to create the same amount of elastic strain. Untreated A shows a significantly lower slope of the Hooke’s curve in comparison to untreated C followed by a viscous-plastic deformation behavior up to fracture.

Figure 2a–c shows the process of deformation of A during micro tensile testing. At the beginning of testing a slight deformation of A is observed. During further process, a constriction towards the middle of the sample occurs and deepens until breakage. On the basis of the sample composition a thickening of the material occurs.

To study the impact of RGX and layering on the mechanical behavior, we investigated the material properties like Young’s modulus for all. Due to the large mechanical mismatch, statements about other parameters such as elongation at break and ultimate tensile strength were accessible for untreated A, A_RGX_, AA and AAA.

**Figure 3.**
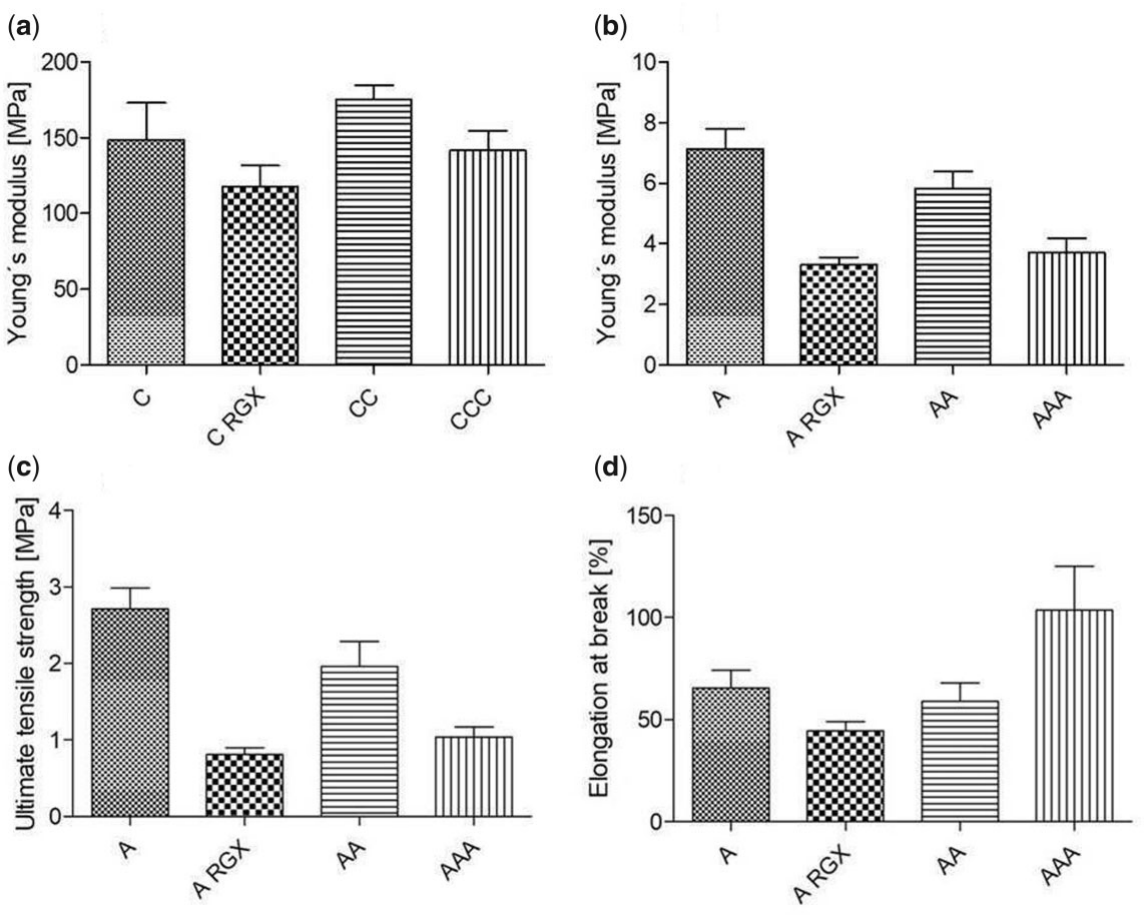
Young’s modulus of untreated and crosslinked collagen single sheets and laminates of C **(a)** and A **(b)** and ultimate tensile strength **(c)** and elongation **(d)** at break of untreated and crosslinked collagen single sheets and laminates of A.

Young’s modulus is experimentally determined from the slope of the stress–strain curves. Generally, the Young’s moduli (Fig. 3a and b) of C with values >100 MPa were considerably higher than the ones measured for A (<10 MPa). For the crosslinked single sheets, the Young’s modulus was smaller than for the untreated ones. C_RGX_ showed a reduction of around 25% compared to untreated C, whereas reduction is twice as high with 50% for A_RGX_ versus untreated A. An increase of the Young’s modulus due to layering was observed, for both CC and CCC. CC exceeded the untreated C by 17%. The same effect of layering could be stated for AA, which exhibited a 35% higher Young’s modulus than the RGX treated monolayer. The same effect of layering could be stated for AA, which exhibited a 35% higher Young’s modulus than the RGX treated monolayer. When a third collagen layer was added no further increase in the Young’s modulus was observed; neither in AAA nor in CCC.

On the contrary, CCC showed a slight reduction of 10% whereas the measured values of AAA were 48%, smaller than those of the untreated ones. The Young’s modulus of AAA was only 6% higher than that of the RGX monolayer.

In the measurement of the ultimate tensile strength of A, a reduction of 70% was detected after RGX treatment (Fig. 3c). After layering, the difference to A untreated was reduced. Compared to A_RGX_, the ultimate tensile strength of bilayer laminates increased by 42%. No additional increase of ultimate tensile strength was detected by addition of a third collagen layer. The ultimate tensile strength of AAA was again reduced and showed a 9% higher value than the A_RGX_ monolayer.

The elongation at break of RGX treated A was reduced by 32% compared to the unmodified A (Fig. 3d). By layering an increase in the ductility was measured. Increasing the number of layers increased the elongation at break. The value of the elongation at break in the AAA multilayer was 60% higher than in the untreated one, indicating a continuous increase with the number of layers.

### Impact of crosslinking and layering on swelling degree

**Figure 4.**
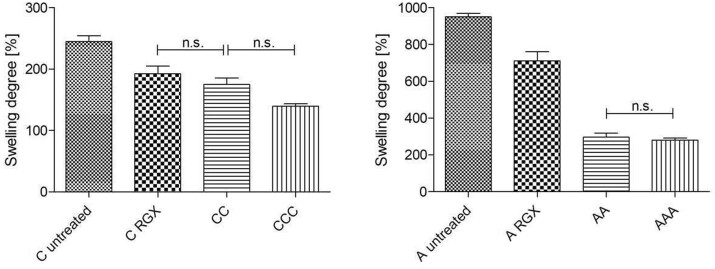
Swelling degree of untreated and crosslinked collagen single and laminate scaffolds (*P < 0.05; n.s. not significant).

To analyze the relationship between the swelling behavior and crosslinking as well as layering, the swelling degree was determined (Fig. 4). Since mostly all of the results were significant only not significant data were highlighted.

The swelling degree of untreated A was more than 3 times higher than that of untreated C. After crosslinking of the collagen sheets, a decrease in water uptake was detected in both materials. While in C_RGX_ no significant differences between the monolayer and the bilayer existed, a significant reduction of water uptake between A_RGX_ and both bi- and multilayer of around 630% was detected. Equal to A, the swelling degree of CC and CCC did not differ from each other.

### Impact of destabilization due to cell contact on mechanical stability

To assess the effect of cells on the destabilization of collagen single sheets (untreated and RGX treated) as well as laminates, the thickness was measured under defined load. A decreased thickness value after 72 h of cell contact compared to 24 h would imply a reduction in the mechanical stability. Table 2 shows all tested combinations of mono-, bi- and multilayers. All samples were fabricated at least in duplicates and for every sample a minimum of five measurements was performed.

**Table 2.** Used collagen samples for thickness analysis

Monolayer	Bilayer	Multilayer
A _(untreated)_	AA	AAA
A _(RGX)_	CC	CCC
C _(untreated)_	AC	CAC
C _(RGX)_		ACA _(unstable)_ no measurement

**Figure 5.**
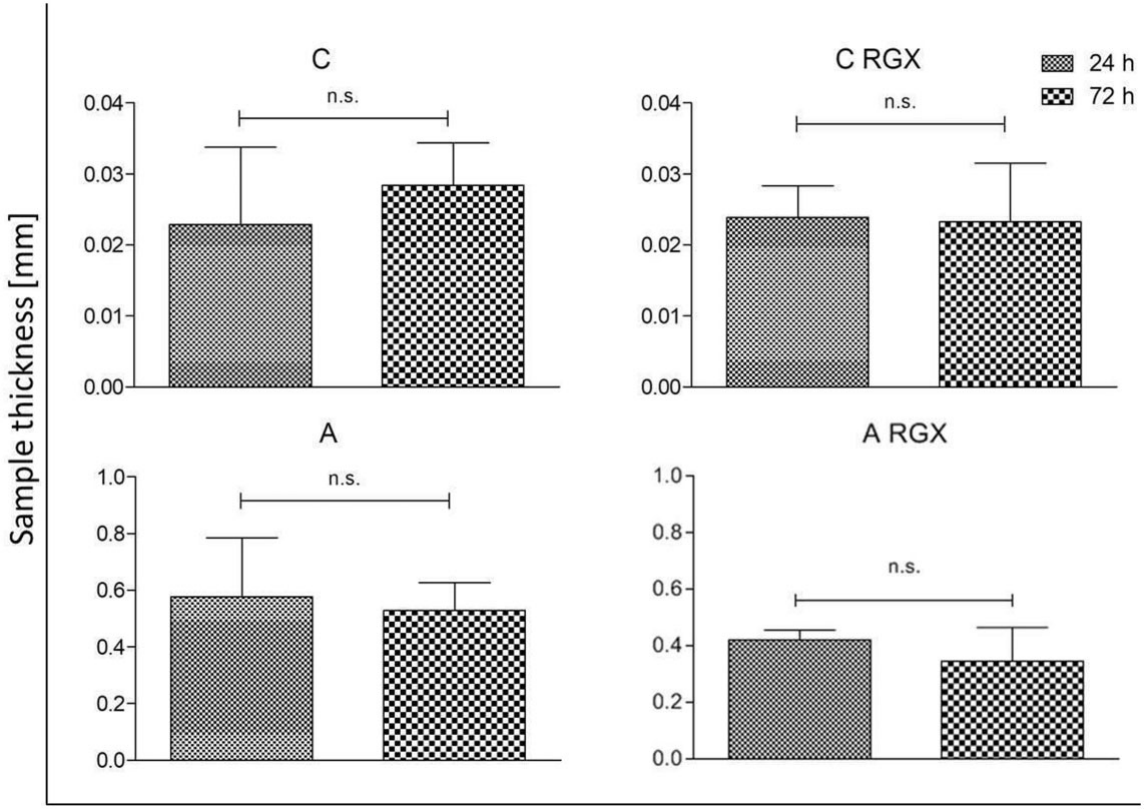
Sample thickness of monolayer collagens after contact with NHDFs for 24 and 72 h (*P < 0.05; n.s. not significant).

No significant differences in thickness were observed between the 24 and 72 h samples of untreated collagen monolayers as well as RGX crosslinked ones (Fig. 5).

**Figure 6.**
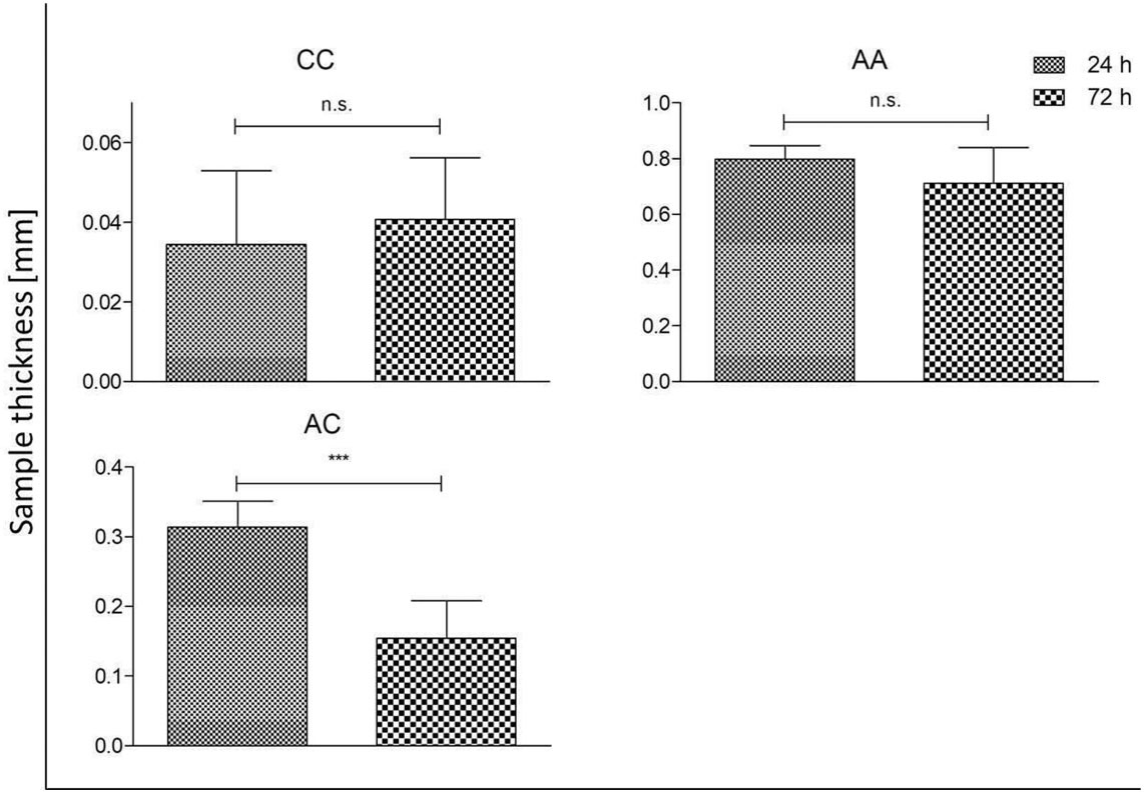
Sample thickness of bilayer collagens after contact with NHDFs for 24 and 72 h (*P < 0.05; **P < 0.01; ***P < 0.001; n.s. not significant).

The bilayer laminates (Fig. 6) CC and AA showed no thickness reduction 72 h after cell contact compared to the 24 h values. A significant effect of the cells on the combined AC laminates with a 40–50% reduction of thickness 72 h after cell contact was observed.

**Figure 7.**
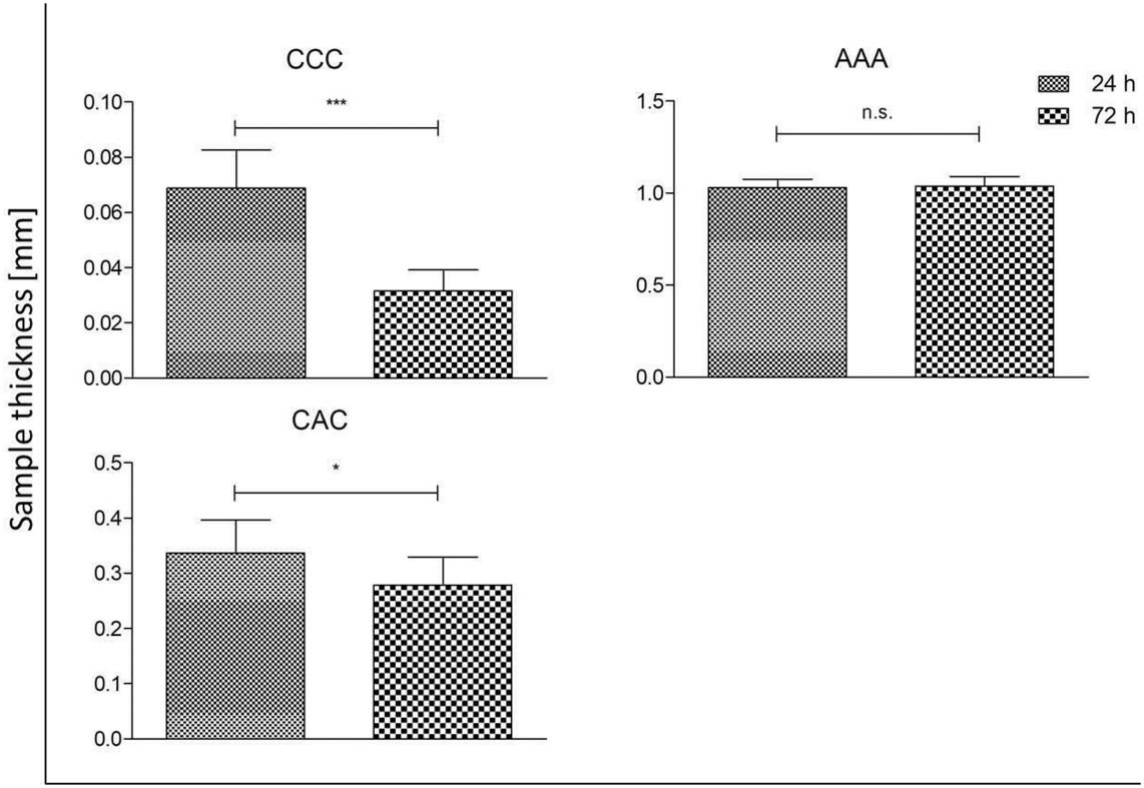
Sample thickness of multilayer collagen laminates after contact with NHDFs for 24 and 72 h (*P < 0.05; **P < 0.01; ***P < 0.001; n.s. not significant).

The thickness of multilayer laminates after cell treatment is shown in Fig. 7. ACA was excluded due its poor stability during removal from the 48-well plate. No decrease in height was observed when cells were incubated for 72 h compared to the values after 24 h on the AAA laminate. For CCC and CAC a significant decrease in thickness was measured. CAC showed a slight reduction of up to 10% whereas the reduction in height of CCC was more than 3 times higher (30–40%).

### Cell viability on collagen laminates

**Figure 8.**
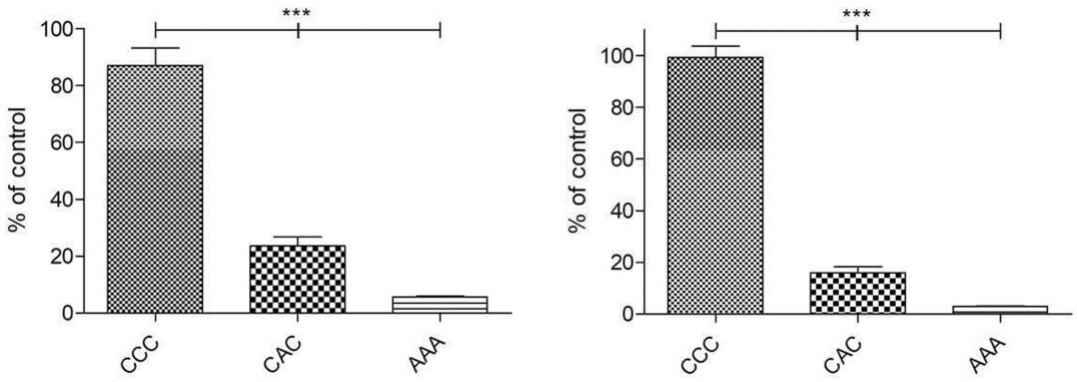
Proliferation of NHDFs on collagen laminates for 24 h (left) and 72 h (right). The results are presented in percentage of the control without collagen sheets (100%) (*P < 0.05; **P < 0.01; ***P < 0.001; n.s. not significant).

To test whether there is a relationship between laminate destabilization and cell proliferation the alamarBlue^®^ assay was used with a similar experimental setup regarding cell number and test time (24 and 72 h) in thickness measurement. Results are presented as percentage of the control (cell culture plate) (Fig. 8). For all used laminates, the proliferation differed significantly from each other. With a viability of 87% on Day 1 and 96% on Day 3, fibroblasts proliferated on CCC as good as on the cell culture surfaces. Hardly any proliferation occurred when cells were seeded on CAC and AAA whereas initial proliferation from Day 0 to Day 1 was higher on CAC.

### *In-vitro* cytotoxicity testing of the laminates

**Figure 9.**
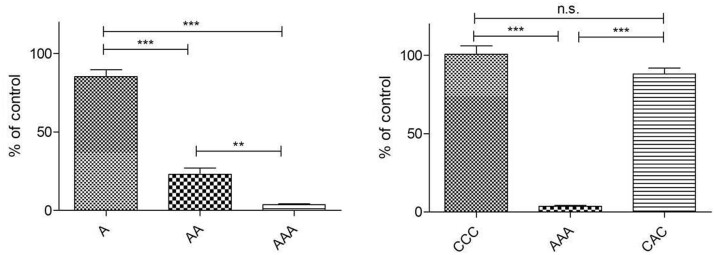
*In-vitro* cytotoxicity testing of the laminates. The results are presented in percentage of the control (medium without collagen) (*P < 0.05; **P < 0.01; ***P < 0.001; n.s. not significant).

For better understanding the proliferation behavior of NHDF’s on the different laminates an *in-vitro* cytotoxicity test with L929 cells was performed (Fig. 9). These are standard cells for this kind of test (DIN 10993-5). Results are presented as percentage of the control (culture medium). Since the medium that was in contact with CCC showed no cytotoxicity, mono- and bilayers were excluded here (see Supplementary Material). A reduction of 87% in cell viability was measured for medium incubated with A_RGX._ Therefore bi and multilayers were also investigated. Viability of the cells decreased with every further layer. For AAA, a viability of 3% was measured which indicated a high toxicity of the medium that had been in contact with these laminates since the DIN 10993-5 defines a reduction of > 30% in cell viability as cytotoxic. No cytotoxic effect was determined for media that had been incubated with CCC and CAC with viability values > 70%. Nevertheless, a negative trend was observed when CCC (100% viability) was compared with CAC (88%).

### Activity of different biomolecules after crosslinking

Since the overall goal of the characterized laminates is to improve healing through the incorporation of different biomolecules, activity tests were performed to investigate the influence of adsorption, crosslinking and release on the functionality of biomolecules. Therefore, we used SDF-1α and BMP-7 and analyzed if crosslinking affected their biological activity.

#### Activity of SDF-1α

**Figure 10.**
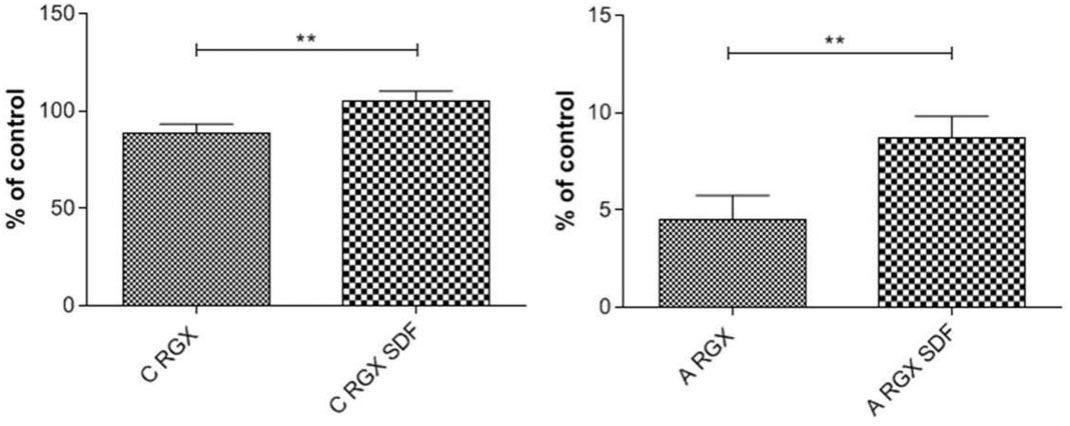
Activity testing of SDF**-**1α after collagen crosslinking through proliferation assays with HUVECs. The results are presented in percentage of the control (culture plate) (*P < 0.05; **P < 0.01; ***P < 0.001; n.s. not significant).

To test the effect of RGX on the biological activity of SDF-1α 500 ng were adsorbed to the collagen sheets and crosslinking was performed as described before. HUVECs were seeded onto the sheets and their proliferation was tested. Results are presented as percentage of the control (culture plate). As demonstrated in Fig. 10, HUVECs showed a significant higher proliferation rate after incubation on both collagen sheets containing SDF-1α. The results confirm that adsorption and subsequent RGX treatment have no negative effect on the bioactivity of SDF-1.

#### Activity of BMP-7

**Figure 11.**
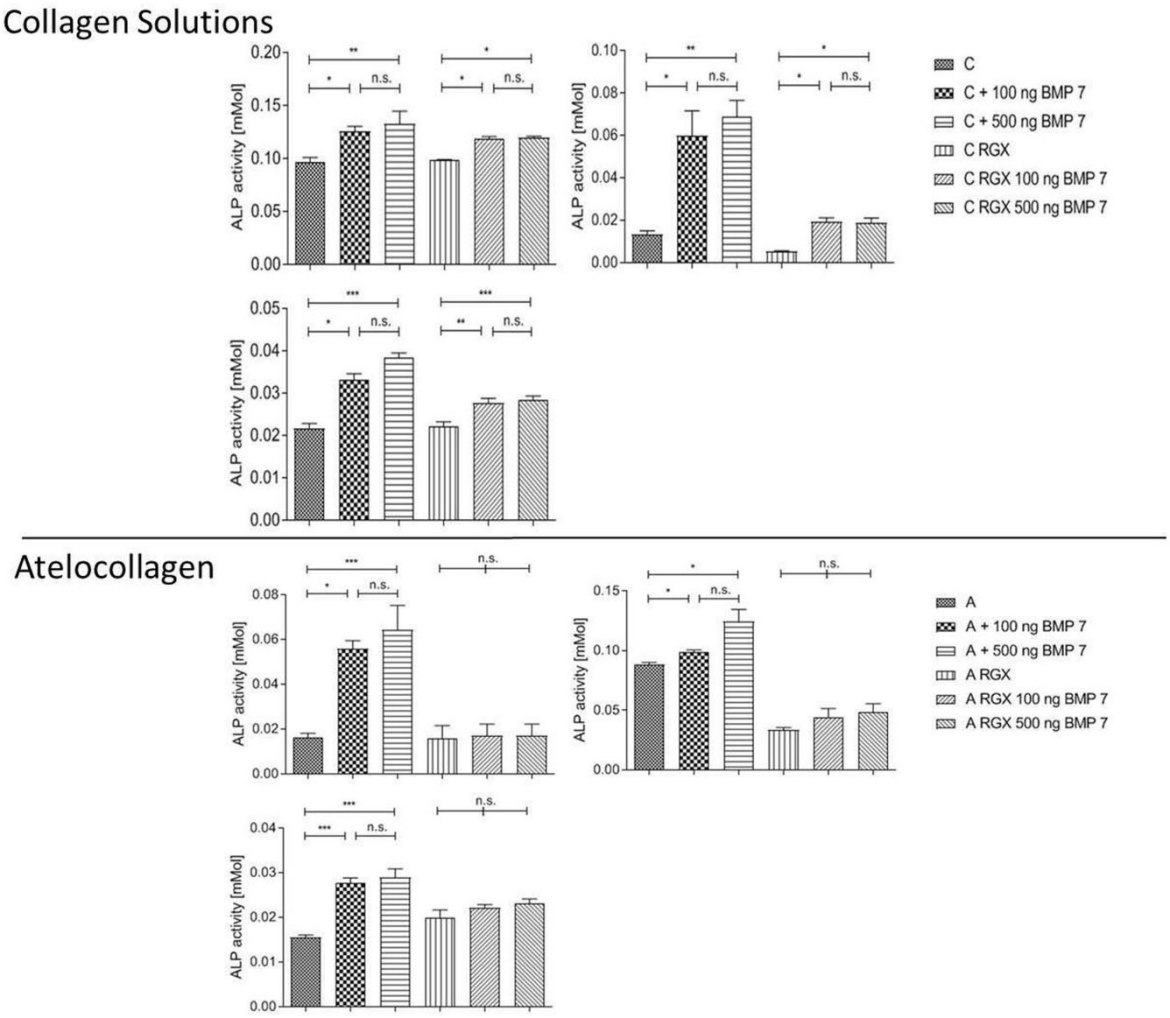
Activity testing of BMP-7 after crosslinking on C and A through an ALP assay. Each diagram represents a single donor (*P < 0.05; **P < 0.01; ***P < 0.001; n.s. not significant).

To test the effect of RGX on the biological activity of BMP-7 100 or 500 ng BMP-7 were adsorbed to the collagen sheets and crosslinking was performed as described before (Fig. 11). Osteoblasts were seeded onto the sheets and their mineralization capacity was investigated with the ALP Assay. Since for BMP-7 on A, after crosslinking no activity was measured the results for BMP-7 adsorbed on the collagen without crosslinking are also presented.

No significant higher ALP activity was measured when the values of 500 ng BMP-7 were compared with that of 100 ng, neither for C nor for A. This finding was independent from collagen treatment (crosslinked or untreated). When BMP-7 was adsorbed on A and C without crosslinking a significant increase in the ALP activity was measured. The same observations were made when BMP-7 was crosslinked with C. For BMP-7 crosslinked in A, no increase in ALP activity was measured even though a positive trend was observed.

## Discussion

Photochemical crosslinking with RB and green light is an excellent method to improve the stability of collagen. By layering collagen, it is possible to tailor the mechanical properties. Therefore we investigated the influence of crosslinking on different collagens and laminate structures regarding their mechanical properties, biomaterial–cell interactions and cell viability.

### Impact of crosslinking on surface topography

To get an overview of the surface topography of the used collagens and the influence of crosslinking by RGX treatment CLSM was performed. CLSM showed that the height profile of both used collagens differed widely. The compact structure of C was also visible in the height profile whereas A showed a sponge-like structure with clear differences in height. Although the structure of A is sponge-like with lots of pores, a declaration regarding the pore interconnectivity is not possible. Diminished differences in height profile of C and a loss of the porous structure of A were visible after RGX treatment. These observations are in line with previous results which showed a total collapse of the structure after contact with PBS [9].

### Impact of crosslinking and layering on mechanical behavior

Micro tensile testing was performed to analyze the biomechanical properties of the collagen scaffolds and their changes after crosslinking and production of laminates. To imitate the environment in the body measurements took place in a wet state. Since the overriding goal is the production of multilayer laminates, the mono and bilayer laminates represent a preliminary test to get a better understanding of the effect of layering on the mechanical behavior.

A high resolution at low forces is necessary for a reliable determination of the Young’s modulus which is a measure for the stiffness of the newly developed collagen material. At the same time, due to the limitation of the load cell with a maximum of 1000 mN a declaration regarding the ultimate tensile strength and elongation at break of C was not possible. As quantitative results for A could be raised, differences in the biomechanical properties of A and C are clearly highlighted.

The ultimate tensile strength describes the maximum stress that a material can withstand during testing. When uncrosslinked monolayers were compared with RGX-treated monolayers a reduction in the ultimate tensile strength was measured. Comparing bilayer laminates with RGX-treated monolayers an increase in the values was observed. However, a further increase of ultimate tensile strength in multilayer laminates was only hypothetical and did not occur. In crosslinked samples compared to untreated ones a decrease in the elongation at break was observed. The greater the elongation at break, the more ductile is the material before rupture takes place; that is the more plastic deformation it can withstand. Unlike the ultimate tensile strength, a continuous increase of the elongation at break was detected from collagen bi- and trilayers. The values for the multilayer laminates were even higher than the values for the untreated ones.

The reduced elongation at break after crosslinking of collagen scaffolds is in accordance with observations of Chan et al. Photochemically crosslinked collagen gels showed a significantly reduced elongation at break compared to the control group. They showed an increase in the ultimate tensile strength whereas our results demonstrated a decrease [3]. Angele et al. showed that highly crosslinking equine and bovine collagen scaffolds with EDC/NHS resulted in a reduction in rupture strength [1]. However, since the experimental conditions, as well as the used collagens and crosslinking methods were different it is difficult to compare the results. Our results clearly demonstrate that it is possible to adapt the mechanical properties through layering collagen sheets for a better use in medical applications. The Young’s modulus for C with values >100 MPa was considerably higher than for A (<10 MPa). Taking into account the macroscopic structure these results were expected since C showed a very compact structure whereas A demonstrated a sponge-like rather soft structure. For C and A, a decrease in the Young’s modulus occurred after RGX treatment. An increased Young’s modulus was determined for bilayer laminates. As seen in the values for the ultimate tensile strength, no further rise was observed through an additional collagen layer within the investigated multilayer laminates.

It is assumed that especially C displayed anisotropic behaviors due to its imprints which were visible in the CLSM images. Their direction of restraining for the micro tensile testing could affect the investigations. Depending on the orientation of the imprints as well as the position, they probably strongly influence also the Young’s modulus.

Wertheim et al. showed that the *ex vivo* RGX treatment of rabbit corneas (consisting mainly of collagen) improved the Young’s modulus [31, 32]. The same observations were made after EDC/NHS crosslinking of microfibrillar collagen [33]. These results are not in line with our results. Generally, it has to be kept in mind that different collagen species and their amino acid composition influence the extent of crosslinking [1]. We have shown an altered surface structure after contact with a fluid. It is also possible that crosslinking changes the pore size and therefore different micro-cracks may occur during testing compared to the uncrosslinked ones [34]. Modifications in pore size through crosslinking could also explain the differences in proliferation behavior of cells on crosslinked sheets compared to untreated ones described earlier [9].

Tensile testing displayed a decrease in the value of every characterized parameter when the untreated monolayer was compared to the crosslinked monolayer. Changes in mechanical parameters like the elastic modulus or strength appeared in atomic bindings on collagen scaffold which enabled an alteration in the collagen structure [35]. Nevertheless, the addition of a second collagen layer improved the values. The effects of a third layer were not predictable, sometimes different. Generally, it is possible to adapt the mechanical parameter through layering of collagens. Especially the material mismatch allows a huge bandwidth of mechanical properties. Therefore, according to the respective medical requirements depending on the application field, it is possible to produce the perfect fitting laminate.

### Impact of crosslinking and layering on swelling degree

Swelling degree was determined to investigate potential correlations between the mechanical characteristics and water uptake. Generally, the swelling degree of A was more than 3 times higher than of C. This result was expected due to the macroscopic sponge-like structure of A and the compact structure of C. After crosslinking of monolayers, the swelling degree was decreased which is in accordance with the literature. A relation between the crosslinking method and the alteration of the swelling degree has been described [11]. Angele et al. showed a decrease in swelling ratio after EDC/NHS crosslinking of collagen scaffolds [1]. They also detected a correlation between dry and wet collagen scaffolds and their mechanical parameters, for example, the elongation at break: This value for example for wet scaffolds was higher than for the dry ones. Transmitting their findings to the amount of water uptake of our bi- and multilayer laminates, we assume that our mechanical values like elongation at break are independent of the water amount. Untreated A had a higher swelling degree and a higher elongation at break compared to the A_RGX_ which is in accordance with the literature [1, 11]. Swelling degree was greatly reduced from monolayer to bilayer but no significant differences in swelling degree for AA and AAA were measured. However, elongation at break constantly increased from monolayer to trilayer, so that the correlation exists only for monolayers, not for multilayers. Another possible support for our thesis that the mechanical values are independent of water uptake are the findings of Steele et al. They wetted their collagen–cellulose composite films with PBS and observed a 70–90% increase in elongation compared to dry films. In case of tensile strength, they demonstrated a decrease when the samples were wet [36]. As mentioned above no differences in swelling degree in AA and AAA were detected in our study, but differences in the ultimate tensile strength were observed. Swelling experiments were performed to investigate potential relations between mechanical properties and water uptake. No clear dependency between swelling degree and mechanical values was observed. Nevertheless for our final aim, the development of collagen laminates with controlled release of biomolecules only the initial uptake of unmodified collagen is relevant, to adjust the volume for biomolecule loading.

### Impact of destabilization due to cell contact on mechanical stability

To investigate the influence of fibroblasts on A and C before and after RGX treatment as well as on the different laminate structure, thickness analyses were performed 24 and 72 h after cell contact. We assumed that a reduction of thickness between the 24 and 72 h samples would indicate a destabilization of the collagens by the cells. No significant differences were observed in the untreated and RGX crosslinked monolayers. In the bilayer laminates, no significant differences in layer thickness were observed for AA and CC. AC laminates showed a significant decrease in layer thickness after 72 h of cell contact. For the multilayer laminates CCC, AAA and CAC, only AAA demonstrated no differences in height. For a better understanding of these findings, proliferation and cytotoxic assays were performed.

We assume that C is very stable due to its compact structure. Based on this fact it might be difficult for cells to degrade the sheets or bilayer laminates. Cells proliferation was best on CCC laminates, which demonstrated a high destabilization rate. Therefore, we assume that the destabilization is not affected by the structure of this collagen film itself, but by the manufacturing method of the laminates. One potential reason for the destabilization of CCC in contrast to CC and C could be the rising number of interfaces. If the edges are not completely and tightly closed cells could migrate between the sheets, proliferate and degrade the collagen from more sides. This is a good characteristic, especially for later medical application.

In all tested samples of A, no impact of the cells on mechanical stability was observed. In context with the viability testing these results are not surprising since the cells hardly proliferated on the sheets. *In-vitro* cytotoxic testing showed a high cytotoxic effect of AAA with a decrease of cytotoxicity through removal of layers. This explains why no changes in mechanical stability were observed. The cytotoxic effect of AAA was independent of the higher RB volume which was used for the Laminate production (data not shown). In contrast to the homogenous laminates, we observed a destabilization of the heterogenous bilayer laminates after cell contact. This can be explained by the combination of the two different collagen sheets with very different characteristics. On C cells proliferate very well and the structure itself is very stable; in contrast to that, A is rather soft and can be easily degraded by cells. In the multilayers CCC and CAC a decrease in height was observed that was stronger in CCC. Under the assumption that A is mechanically weaker than C we would expect that the differences are more pronounced for CAC. However, the cells proliferated much better on CCC than on CAC and the cytotoxic effect of A may slow down collagen degradation. Therefore the destabilization of laminates is not only dominated by the material density itself but also in the potential to promote or inhibit cell proliferation. The reduction in thickness and therefore a destabilization of the scaffold is associated with the higher matrix metalloproteinase-1 (MMP-1) production. This endopeptidase is produced by fibroblasts and is responsible for collagen digestion [37, 38]. Due to better proliferation and therefore higher cell numbers more MMP-1 is produced that degrades collagen more effectively resulting in a reduction in height. The interplay between biomaterials such as collagen and cells in TE scaffolds is highly complex. Many parameters like surface topography, pore size and mechanical strength determine cell proliferation as well as expression of intrinsic factors, for example, matrix metalloproteinase 2 (MMP-2) or ALP [35, 39–46]. In this context, the latter is a specific marker for the early stage of osteogenesis [30]. By constructing mono-, bi- and multilayer laminates, not only the number of interfaces varies, the composition of laminates also changes the mechanical stability, the cell-accessible surface area and the shape. As a consequence, the behavior of the cells changes. Depending on the laminate structure cells get in contact with different collagen surfaces and structures at the outside and inside of the laminate. Different sections of the sheet demonstrate different characteristics, indicating how important it is to characterize these laminates individually. However, it is not easy to determine whether the cell response is dependent on the cell–substrate interactions or the macroscopic structure of the biomaterial [47, 48]. Our results show that destabilization of the laminates (AC, CCC and CAC) occurred after cell contact, indicating collagen degradation. This is the prerequisite for using laminates in medical application as drug carrier and target release, for example, to induce bone regeneration. By incorporating different bioactive molecules in different layers in such a multilayer structure, it is possible to control the release of different molecules. Bioactive molecules in the outer layer are released directly, whereas the molecules incorporated in the inner layer will have delayed release since the outer layer has to be degraded first. Therefore, we assume a slower release for incorporated molecules, especially in the inner layer of CAC compared to CCC because of the higher degree of destabilization in CCC.

This huge bandwidth of mechanical properties enables a wide range of applications in the clinical field. Since different compartments of the body have different requirements, it is possible to tailor the characteristics of the laminates regarding the patients’ needs individually. As a consequence, laminates will improve healing.

### Impact of crosslinking on the bioactivity of SDF-1α and BMP-7

For later medical applications, it is necessary that incorporated biomolecules maintain their bioactivity after collagen crosslinking. Therefore, SDF-1α and BMP-7 were adsorbed on the collagens followed by RGX treatment. SDF-1α was still active after crosslinking on both A and C demonstrated by increased proliferation of HUVECs. BMP-7 was still active after release from C, measured by ALP activity of hOB, whereas no significant higher ALP activity was measured after release from A even though a positive trend was evident. For BMP-7 each diagram represents a single donor since the cells are varying greatly in their ALP expression. Addition of the single donors would lead to a falsification of the results. As the hOBs are primary cells such differences are absolutely normal.

Since SDF-1α and BMP-7 have a positive effect on the cells after crosslinking from C it can be assumed that RGX treatment itself doesn’t influence the bioactivity. One possible explanation for this behavior is the difference in mechanical alterations depending on their initial mechanical properties in A and C after crosslinking. We demonstrate a decrease in swelling degree and in the Young’s modulus when C and A were crosslinked. Furthermore, lower values for the ultimate tensile strength and the elongation at break were measured when A was RGX treated. In this context, the size of the proteins may play a crucial role since BMP-7 is more than 2 times greater than SDF-1α. Structural changes in the biomaterial itself may affect the protein–material interaction. Especially when the tested collagens exhibit obvious differences in their macroscopic structure, huge differences would be expected. For BMP-7 no differences between the concentrations were measured in the *in-vitro* experiments. This enables to work with the lower dose of 100 ng. Therefore it is possible to reduce costs since BMP-7 is very expensive [49]. Nevertheless, more research needs to be done to optimize the needed concentrations especially for SDF-1α as well as for BMP-7. In particular, for later *in-vivo* experiments, it is necessary to get a deeper understanding of the effective concentrations. These results show that RGX treatment is a promising crosslinking method to produce collagen laminates for regenerative medicine since the method itself doesn’t affect the activity of the incorporated molecules.

## Conclusion

To improve tissue regeneration, collagen laminates for the release of bioactive molecules are a promising concept. To tailor the properties of such laminates to a given application, it is necessary to understand the used biomaterial as well as the cell–scaffold interactions fundamentally. Therefore, we investigated two commercial collagens in the unmodified and crosslinked states as well as laminates there of regarding their degradation after cell contact, their mechanical stability and their swelling degree. Our results show that it is possible to adjust the mechanical characteristics by the production of multilayer laminates. With these findings, laminate composition can be adapted to develop scaffolds for tissue regeneration in medical applications.

## Supplementary data

Supplementary data are available at *REGBIO* online.

## Supplementary Material

rbab059_Supplementary_Data
