# Supplementary material for: Mechanical characterization of rose bengal and green light crosslinked collagen scaffolds for regenerative medicine
Source: Regen Biomater. 2021 Nov 2;8(6):rbab059. doi: 10.1093/rb/rbab059 (PMC8633790; doi:10.1093/rb/rbab059)
Supplement: rbab059_Supplementary_Data [file rbab059_supplementary_data.docx]

**Supplementary data**


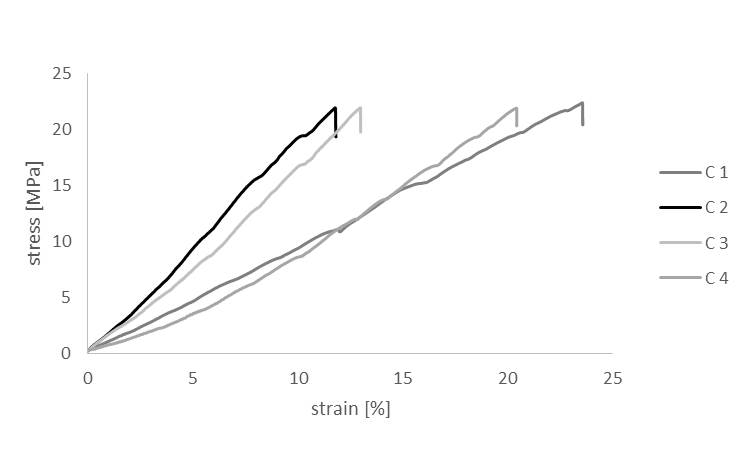


Figure 1: Different curve progressions for micro tensile measurements of C.


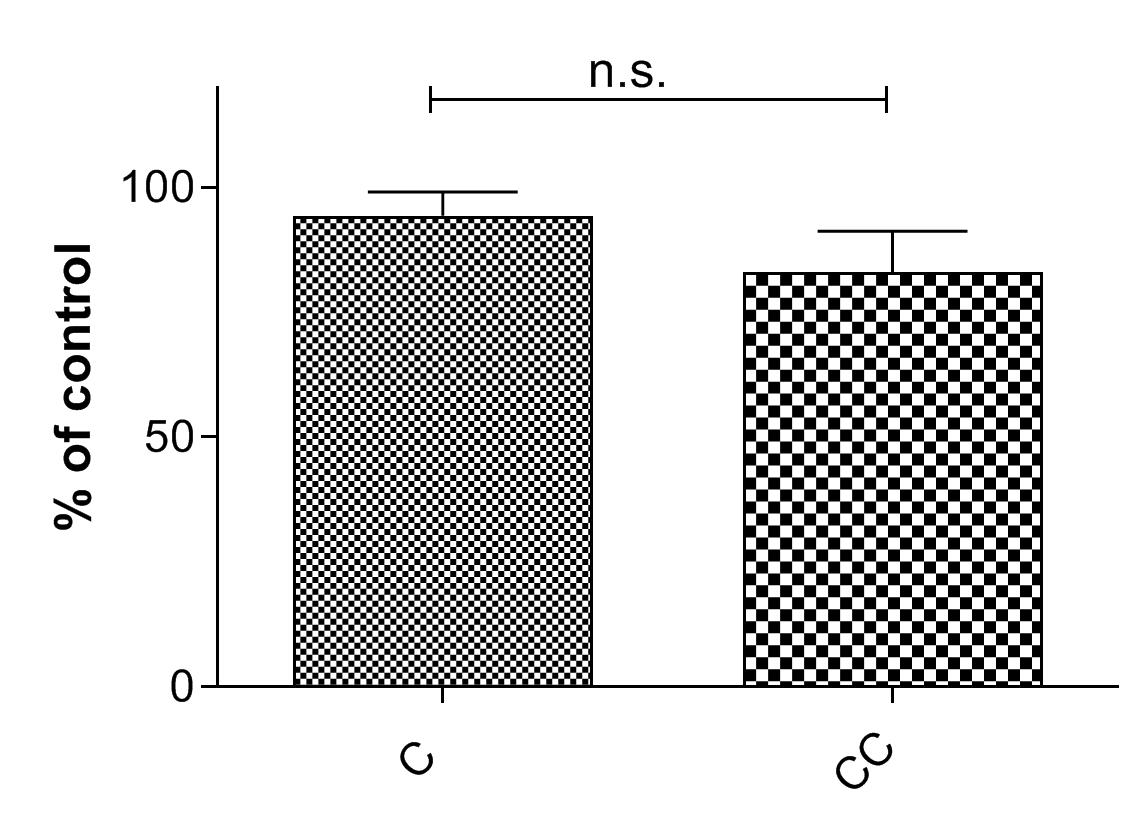


**Figure 2: In-vitro-cytotoxicity testing of the C and CC. The results are presented in percentage of the control (medium without collagen). (* p < 0,05; ** p < 0,01; *** p < 0,001; n.s. not significant).**
